# Effect of Plasma Membrane Cholesterol Depletion on Glucose Transport Regulation in Leukemia Cells

**DOI:** 10.1371/journal.pone.0041246

**Published:** 2012-07-30

**Authors:** Cristiana Caliceti, Laura Zambonin, Cecilia Prata, Francesco Vieceli Dalla Sega, Gabriele Hakim, Silvana Hrelia, Diana Fiorentini

**Affiliations:** Biochemistry Department “G. Moruzzi”, Alma Mater Studiorum-University of Bologna, Bologna, Italy; Faculté de médecine de Nantes, France

## Abstract

GLUT1 is the predominant glucose transporter in leukemia cells, and the modulation of glucose transport activity by cytokines, oncogenes or metabolic stresses is essential for their survival and proliferation. However, the molecular mechanisms allowing to control GLUT1 trafficking and degradation are still under debate. In this study we investigated whether plasma membrane cholesterol depletion plays a role in glucose transport activity in M07e cells, a human megakaryocytic leukemia line. To this purpose, the effect of cholesterol depletion by methyl-β-cyclodextrin (MBCD) on both GLUT1 activity and trafficking was compared to that of the cytokine Stem Cell Factor (SCF). Results show that, like SCF, MBCD led to an increased glucose transport rate and caused a subcellular redistribution of GLUT1, recruiting intracellular transporter molecules to the plasma membrane. Due to the role of caveolae/lipid rafts in GLUT1 stimulation in response to many *stimuli*, we have also investigated the GLUT1 distribution along the fractions obtained after non ionic detergent treatment and density gradient centrifugation, which was only slightly changed upon MBCD treatment. The data suggest that MBCD exerts its action *via* a cholesterol-dependent mechanism that ultimately results in augmented GLUT1 translocation. Moreover, cholesterol depletion triggers GLUT1 translocation without the involvement of c-kit signalling pathway, in fact MBCD effect does not involve Akt and PLCγ phosphorylation. These data, together with the observation that the combined MBCD/SCF cell treatment caused an additive effect on glucose uptake, suggest that the action of SCF and MBCD may proceed through two distinct mechanisms, the former following a signalling pathway, and the latter possibly involving a novel cholesterol dependent mechanism.

## Introduction

Malignant cells have been shown to utilize more glucose than normal cells *in vitro* and *in vivo*. These cells exhibit increased rates of glucose uptake mediated by facilitative glucose transporter (GLUT) proteins. Among these, the GLUT1 isoform is frequently overexpressed and many studies suggest that GLUT1 expression may be of prognostic significance [1], [2]. GLUT1 is the predominant glucose transporter in hemopoietic cells, and cytokines regulate glucose uptake through modulation of GLUT1 protein levels and cell surface trafficking [3]. Hence, maintenance of glucose transport by cytokines, oncogenes or metabolic stresses appears to be an essential feature of the survival response of hemopoietic cells. However, the molecular mechanisms allowing these molecules or conditions to control GLUT1 trafficking and degradation, are still under debate.

An acute increase in the V_max_ for glucose uptake occurs in many GLUT1-expressing cell types after exposure to a variety of *stimuli*. This early response is associated with no increase in the total amount of cell glucose transporter and could result from a different GLUT1 distribution between intracellular storage pools and the cell surface, as observed in insulin-dependent cells, or by an unmasking activation of GLUT1 molecules already resident in the plasma membrane [4]. Data from the literature demonstrated that cytokine stimulation promotes GLUT1 activity and trafficking through phosphatidylinositol 3-kinase (PI3K) and its downstream effector Akt, both in a murine lymphoid/myeloid hemopoietic precursor cells [3] and in human embryonic kidney cells [5]. We have previously shown that a growth factor such as Stem Cell Factor (SCF) activates glucose transport through a translocation of GLUT1 protein from intracellular stores to cell membrane in the human hemopoietic cell line M07e expressing only GLUT1 and that this effect can be mimicked by exogenous H_2_O_2_ in a PI3K-independent way [6].

PI3K pathway and activation of Akt play a well established role in GLUT4 vesicle trafficking to the cell membrane in response to insulin [7], but emerging evidence suggests that a second signalling cascade, that functions independently of the PI3K pathway, is also required for this process in 3T3-L1 adipocytes. This second pathway involves the G-protein TC10, which functions within the specialized environment of lipid raft microdomains at the plasma membrane [8]. Moreover, a relationship between plasma membrane cholesterol and GLUT4 levels has recently become apparent and it has been observed that the recruitment of intracellular GLUT4 to the plasma membrane is achieved by moderate depletion of membrane cholesterol obtained in different ways [9], [10]. Regulation of GLUT4 translocation by moderate cholesterol loss did not involve known insulin-signalling proteins, but could proceed *via* a novel cholesterol-dependent mechanism [9], [10]. These new findings are in agreement with the marked sensitivity to the lipid environment observed for GLUT1 isoform, which has been described in the past [11].

Recently, GLUT1 transporters were reported to be localized in part to detergent-resistant membrane domains (DRMs) in a number of cell types [12] and Barnes et al. hypothesized a role for lipid rafts in GLUT1 stimulation observed in Clone 9 cells in response to stress [13]. In fact, recent studies have indicated that lipid rafts or lipid microdomain platforms may be implicated in signalling and membrane trafficking of a variety of cells in response to agonists or *stimuli* [14], [15].

In light of these reports and in order to better elucidate the mechanism by which SCF activates GLUT1 translocation in M07e cells, in the present study we investigated whether plasma membrane cholesterol depletion play a role in glucose transport activity of this cell line. To this purpose, we compared the effect of methyl-β-cyclodextrin (MBCD), the most efficient compound used to induce cholesterol depletion from plasma membrane, and SCF on both GLUT1 activity and trafficking in M07e cells. In parallel, the effect of MBCD on GLUT1 distribution in the plasma membrane fractions was probed by density-gradient centrifugation of detergent-treated cell lysates. Data obtained in this study show that the reduction of plasma membrane cholesterol content significantly affects GLUT1 activity and that MBCD exerts its action *via* a cholesterol-dependent mechanism that ultimately results in augmented GLUT1 translocation. Unlike SCF, the MBCD effect does not involve Akt and phospholipase Cγ (PLCγ) phosphorylation.

## Materials and Methods

### Chemicals

Iscove’s modified Dulbecco’s medium (IMDM) and foetal calf serum (FCS) were purchased from BioWhittaker (Walkersville, MD, USA). Interleukin-3 (IL-3) was from Invitrogen (Carlsbad, CA, USA), SCF was provided by Immunological Sciences (Società Italiana Chimici, Rome, Italy). Methyl-β-cyclodextrin (MBCD), nystatin, phloretin, 2-deoxy-D-glucose (DOG), cholesterol, phenylmethylsulfonyl fluoride (PMSF), N-tosyl-L-lysine chloromethyl ketone (TLCK), N-tosyl-L-phenylalanine chloromethyl ketone (TPCK), sodium orthovanadate, protease inhibitor cocktail, Trypan blue solution (0,4%), 3-(4,5-dimethylthiazol-2-yl)-2,5-diphenyl tetrazolium bromide (MTT), Igepal, mouse monoclonal antiserum against tubulin, rabbit antibody against flotillin-2 were from Sigma-Aldrich (St. Louis, MO, USA). 2-deoxy-D-[2,6-^3^H]-glucose and [1,2-^3^H(N)]-Cholesterol were from PerkinElmer (Massachusetts, USA); nitrocellulose paper, ECL Plus Western Blotting Detection Reagents were from GE Healthcare (UK). Triton-X-100 and sucrose were from Merck (Whitehouse Station, NJ, USA). DC Protein Assay Kit were from Bio-Rad (USA). Anti-transferrin receptor (CD71) was provided by BD Biosciences (San Jose, CA, USA); anti-Lyn antibody was from Abcam (Cambridge, UK). Anti-GLUT1 (# CBL242) and anti-p-tyrosine (# 06–427) were from Millipore (Temecula, CA, USA). Anti-GLUT1 (N-20), fluorescent FITC-conjugated anti-goat IgG and anti-PLCγ1 were from Santa Cruz Biotechnology (Santa Cruz, CA, USA). Anti-Akt, anti-p-Akt, anti-ERK 1/2, anti-p-ERK 1/2 were from Cell Signalling Technology (Beverly, MA, USA). Sulfosuccinimidyl 6-(biotinamido) hexanoate (NHS-LC-biotin), streptavidin-agarose beads were purchased from Pierce (Rockford, IL, USA).

All the other chemicals and solvents were of the highest analytical grade.

### Cell Culture

M07e, purchased from DSMZ, Braunschweig (Germany), is a human leukemia megakaryocytic cell line whose proliferation is IL-3 or GM-CSF dependent. Cells were cultured in IMDM supplemented with 5% foetal calf serum and 10 ng/mL IL-3 as previously reported [16]. The experimental model employed 16–24 h IL-3-starved cells, as these conditions were more apt for focusing experiments on cholesterol role, ruling out other growth factor effects.

### Cell Viability Evaluation

Viable cells were evaluated by the Trypan blue exclusion test. Cell viability was also assayed by the MTT assay [17], since the reduction of tetrazolium salts is widely accepted as a reliable way to examine cell viability/proliferation. Cells were incubated with 0.5 mg/mL MTT for 4 h at 37°C. At the end of the incubation, purple formazan salt crystals were formed and dissolved by adding the solubilization solution (10% SDS, 0.01 M HCl), then the plates were incubated overnight in humidified atmosphere (37°C, 5% CO_2_). The absorption at 570 nm was measured on a multi-well plate reader (Wallac Victor^2^, PerkinElmer).

### Cholesterol Depletion

M07e cells suspended in culture medium were incubated overnight with [^3^H]-cholesterol (0.5 µCi/mL), then washed, suspended in PBS and exposed to different concentrations of MBCD (2.5–25 mM) for different times (10–40 min). To measure the relative cholesterol content, cells were washed twice in PBS, pelleted at 4000 *g* for 1 min and sample radioactivity was quantified by liquid-scintillation counting. To generate cyclodextrin/cholesterol-inclusion complexes able to donate cholesterol to the membrane [18], [19], 20 mg/mL cholesterol (25 mM final concentration) were dissolved in ethanol and mixed with 10% MBCD in PBS at 37°C. MBCD/cholesterol mixture was used at 1∶25 dilution.

### Glucose Transport Assay

The measurement of glucose transport rate was performed according to [16]. In brief, IL-3-starved cells (4×10^6^/mL) were suspended in PBS, incubated with different *stimuli* and/or inhibitors at 37°C and then treated with a mixture of 2-deoxy-D-[2,6-^3^H] glucose (0.4 µCi/assay) and 1 mM unlabeled glucose analogue (DOG mixture) for 2 min at 37°C under conditions where the uptake was linear at least for 20 min. After this time, the uptake was stopped by adding phloretin (final concentration, 0.2 mM), a potent inhibitor of glucose transport activity. Sample radioactivity was measured by liquid scintillation counting. Transported 2-deoxy-D-glucose was less then 20% of the extracellular-sugar concentration, therefore glucose transport assay could be considered in *zero-trans* conditions [20]. M07e cells deprived of medium components and suspended in PBS during glucose transport measurements maintained their viability up to 2 hours at 37°C, thus the number of viable cells during time intervals of experiments was considered constant (data not shown).

To test the effect of MBCD on the glucose transport activity, cells were incubated at 37°C with 10 mM MBCD for 20 min, washed, re-suspended in 0.5 mL of PBS and added with DOG mixture for the measurement of glucose uptake as previously described.

To test the effect of nystatin, an endocytosis inhibitor [21], IL-3 starved cells suspended in culture medium were incubated with 50 µg/mL nystatin at 37°C for 3 hours. Cells were washed and re-suspended in PBS at 37°C, incubated or not with 10 mM MBCD for 20 min, washed, re-suspended in PBS and assayed for glucose transport activity.

### Preparation of Lipid Rafts/Caveolae

Lipid rafts and detergent-soluble proteins were separated by flotation assays adapted from previously described methods [22]. 200×10^6^ M07e cells (approx. 6 mg of protein) were washed twice with PBS, pelleted at 300 *g* for 7 min and left on ice for 10 min. The cell pellet was incubated at 4°C in 1.2 mL of lysis buffer (1% Triton X-100, 150 mM NaCl, 50 mM TRIS and 5 mM EDTA supplemented with 0.1 mM PMSF, 0.1 mM TLCK, 0.1 mM TPCK, 1 mM orthovanadate and protease inhibitor cocktail, pH 8.0). In all subsequent steps, solutions and samples were kept at 4°C. The lysates were then spun for 10 min at 6000 *g* in an Eppendorf Microfuge and supernatants were homogenized in a Potter homogenizer with 20 strokes. For sucrose gradient separations, 1.0 mL of 80% sucrose prepared in PBS was mixed with an equal volume of homogenized sample and then overlaid with a 5–40% sucrose linear step gradient (1.3 mL each of 5%, 30% and 40% sucrose in PBS). After centrifugation in a SW50.1 Beckman rotor at 160.000 *g* for 18 h at 4°C, nine 500 µL fractions were collected from the top of the gradient. Same volume aliquots of each fraction were added with Laemmli buffer containing both mercaptoethanol and bromophenol blue and boiled for 3 min. Samples were then subjected to SDS-PAGE and immunoblotting.

To measure the relative cholesterol content along the sucrose gradient fractionation, M07e were pre-incubated at 37°C for 16 hours with [^3^H]-cholesterol (0.1 µCi/mL) in cell culture medium. Cells exposed (or not) to 10 mM MBCD for 20 min were lysed with TX-100 at 4°C and subjected to sucrose gradient centrifugation as previously described. [^3^H]-cholesterol content of each of the nine fractions collected was quantified by liquid scintillation counting.

### Biotinylation of Plasma Membranes

Plasma membrane biotinylation was performed as previously described [6]. Briefly, M07e cells (40×10^6^ cells) suspended in PBS were incubated with or without 10 mM MBCD for 20 min, washed twice in ice-cold PBS, pH 8.0, then suspended in 10 mL of cold biotinylation buffer (120 mM NaCl, 30 mM NaHCO_3_ and 5 mM KCl, pH 8.5) containing 0.3 mg/mL freshly added NHS-LC-biotin. After 30 min of gentle swirling at 4°C, cell suspensions were centrifuged and the pellets were washed three times with 10 mL of buffer containing 140 mM NaCl, 20 mM Tris and 5 mM KCl, at pH 7.5. Cells were then lysed in 1.3 mL of hypotonic homogenisation buffer containing 10 mM NaHCO_3_ and 100 µM concentration each of TPCK, TLCK and PMSF. After 10 min on ice, cells were homogenised in a Potter homogenizer with 20 strokes and 130 µL of buffer containing 1.5 M NaCl and 100 mM Tris (pH 7.0) were added. The homogenates were then spun for 15 s at 18000 *g* in an Eppendorf Microfuge to sediment nuclei. The resulting post-nuclear supernatants were added to 1.5 mL Eppendorf tubes containing 50 µL of streptavidin-agarose beads that had been sedimented following pre-equilibration with 1 mL of homogenization buffer, and 5 µL of 20 mM PMSF were added to each mixture. After gentle mixing of the samples by repeated inversion at 4°C for 30 min, the beads were pelleted and washed three times with 1 mL of homogenisation buffer containing freshly added protease inhibitors. The final pellets were re-suspended in 80 µL of 1.2×Laemmli buffer and incubated at 65°C for 30 min. The beads were pelleted and the supernatants containing solubilized plasma membrane were removed and frozen overnight prior to use. Cell fractions containing equal amounts of protein were added with 0.006% bromophenol blue and 4% β-mercaptoethanol (final concentrations) and boiled for 3 min. Samples were then subjected to SDS-PAGE and immunoblotting.

### Protein Assay

Protein concentration was usually determined by the Bradford method with BSA as standard [23].

The protein content of fractions obtained from sucrose gradient centrifugation or after biotinylation was determined by a Bio-Rad DC protein assay kit, using BSA in the presence of appropriate concentration of Triton X-100 or SDS as a standard.

### Immunofluorescence

M07e cells (2×10^6^) were incubated for 20 min with or without 10 mM MBCD, then pelleted and fixed in 3% (w/v) paraformaldheyde for 15 min. Cells were washed twice with HBSS, blocked with PBS/BSA 1% (w/v) for 1 hour and then incubated for 1 hour with 20 µg/mL of anti-GLUT1 raised against a peptide within an extracellular domain of the human transporter protein. Cells were then treated for 1 hour with fluorescent FITC-conjugated rabbit anti-goat IgG, mounted on slides and visualized using an Olympus IX50 microscope.

### SDS-PAGE and Western Blot Analysis

For the study of the phosphorylation cascade induced by SCF or MBCD, cells were lysed with buffer (1% Igepal, 150 mM NaCl, 50 mM Tris-HCl, 5 mM EDTA, 0,1 mM PMSF, 0,1 mM TLCK, 0,1 mM TPCK, 1 mM orthovanadate and protease inhibitor cocktail, pH 8.0) in ice for 15 min. Cell lysates or fractions obtained after sucrose gradient centrifugation or biotinylation were separated on 10% SDS-polyacrylamide gel using a Mini-Protean II apparatus (BioRad Laboratories) and then transferred electrophoretically to nitrocellulose membranes. Nonspecific binding to membrane was blocked by incubating in Tris-buffered saline (TBS)/Tween, pH 8.0, containing 5% nonfat dried milk for 1 hour at room temperature. Blots were probed overnight at 4°C with primary antibodies, washed with TBS/Tween and then incubated for 1 hour at room temperature with secondary horseradish peroxidase conjugates antibodies. Membranes were washed and the antigens were then visualized by addition of ECL Plus Western Blotting Detection Reagents.

### Immunoprecipitation

M07e cells were incubated in PBS with SCF or MBCD as previously described and cell lysates were prepared as described above for Western Blot. Lysates containing equal protein amounts were incubated overnight with 2 µg affinity-purified monoclonal anti-p-tyrosine antibody. Then samples were incubated with protein A-Sepharose beads for 1.5 h at 4°C and then pelleted. Pellets were washed four times with lysis buffer, treating with reducing buffer containing 4% β-mercaptoethanol and then boiled for 3 min. Samples were then subjected to SDS-PAGE and immunoblotting.

### Statistical Analysis

Results are expressed as means with standard deviation. Differences between the means were determined by two-tailed Student’s *t* test or by Newman-Keuls multiple comparison test following one-way ANOVA and were considered significant at *P*<0.05.

## Results

### Setting of Cholesterol Depletion Conditions

**Figure 1:**
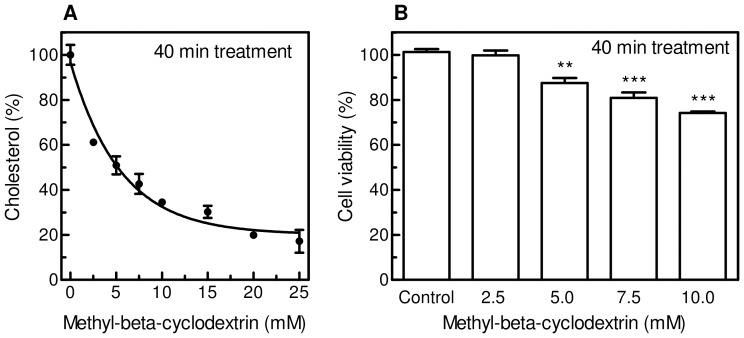
Effect of different MBCD concentrations on cholesterol content and cell viability of M07e cells. (**A**) M07e cells were incubated with [^3^H]-cholesterol (0.5 µCi/mL) in cell culture medium for 16 h at 37°C, washed, re-suspended in PBS and treated with MBCD for 40 min at the concentrations indicated. Cell suspensions were washed with PBS, then [^3^H]-cholesterol content was estimated by liquid scintillation counting. (**B**) The viability of the cells treated as described in Fig. 1A was evaluated by Trypan Blue exclusion test. Results are expressed as means ± SD of three independent experiments, each performed in triplicate. **P<0.005, significantly different from control cells; ***P<0.0005, significantly different from control cells.

**Figure 2:**
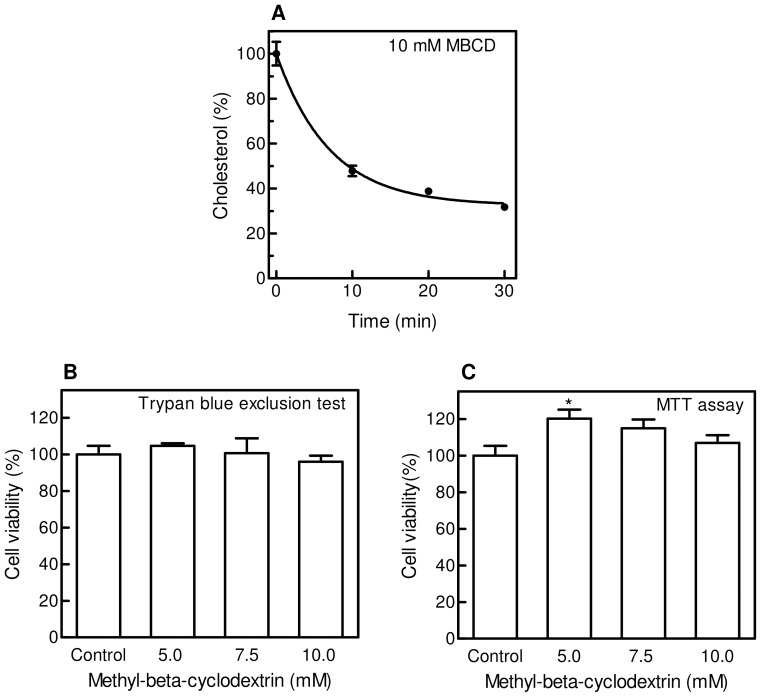
Effect of 10 mM MBCD on cholesterol content and cell viability/proliferation of M07e cells. (**A**) M07e cells were incubated with [^3^H]-cholesterol (0.5 µCi/mL) in cell culture medium for 16 h at 37°C, washed, re-suspended in PBS and treated with 10 mM MBCD at the times indicated. Cell suspensions were washed with PBS, then [^3^H]-cholesterol content was estimated by liquid scintillation counting. (**B**) Cells treated as described in Fig. 2A, were exposed to different MBCD concentrations for 20 min. Viability was evaluated by Trypan Blue exclusion test. (**C**) Cells treated as described in Fig. 2A, were exposed to different MBCD concentrations for 20 min. Proliferation was evaluated by MTT assay as described in the Materials and Methods section. Results are expressed as means ± SD of three independent experiments, each performed in triplicate. *P<0.05, significantly different from control cells.

Many studies have shown that a variety of cellular functions are affected when cells are exposed to β-cyclodextrins, a class of agents commonly used to remove membrane cholesterol [18]. The degree of cholesterol depletion is a function of the β-cyclodextrin derivative used, its concentration, incubation time and temperature. Furthermore, it may differ significantly between cell types even when comparable β-cyclodextrin concentrations and exposure times are applied [19], [24]. Among the different dextrin derivatives, methyl-β-cyclodextrin (MBCD) was shown to be the most efficient as acceptor of cellular cholesterol, and it is most commonly used. Therefore, we chose methyl-β-cyclodextrin to induce cholesterol depletion from plasma membrane of M07e cells, and performed experiments to set the desired conditions. First of all, M07e cells suspended in culture medium were incubated overnight with [^3^H]-cholesterol (0.5 µCi/mL), then washed and exposed to different concentrations of MBCD (2.5–25 mM) for 40 min (Fig. 1A). Among the treatments able to remove at least 60% of cellular cholesterol, 7.5 and 10 mM MBCD significantly affected cell viability, as reported in Fig.1B. Fig. 2A represents the time course of MBCD effect on cholesterol level, evidencing that 10 mM MBCD for 20 min was able to remove about 60% cholesterol, keeping cell viability almost unchanged (Fig. 2B and C). These conditions established a lipid environment alteration associated with membrane integrity.

### Effect of Cholesterol Depletion on Glucose Uptake in M07e Cells

**Figure 3:**
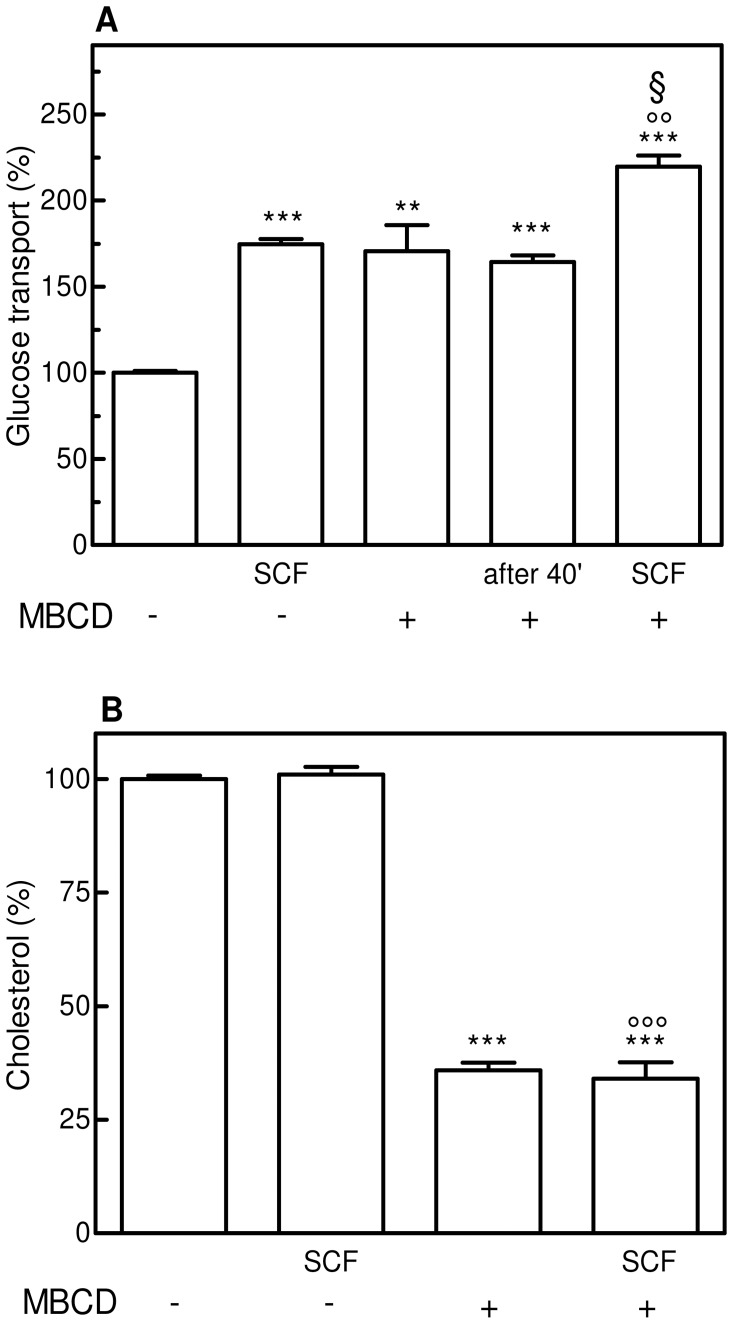
Effect of SCF and/or MBCD on glucose transport and cholesterol content in M07e cells. (**A**) IL-3-starved cells were incubated (or not) in PBS at 37°C with 5 ng/mL SCF for 15 min, then assayed for glucose transport activity as described in the Materials and Methods section. To test the effect of MBCD, cells were incubated in PBS at 37°C with 10 mM MBCD for 20 min, washed, re-suspended in PBS and assayed for glucose transport activity both immediately and after 40 min incubation at 37°C (reported as “after 40′ ”). When subjected to both *stimuli*, M07e cells were incubated with MBCD, washed, treated with SCF and assayed for glucose uptake. (**B**) M07e cells were incubated with [^3^H]-cholesterol (0.5 µCi/mL) in cell culture medium for 16 h at 37°C, washed, re-suspended in PBS and treated (or not) with 10 mM MBCD for 20 min in the presence or absence of 5 ng/mL SCF for 15 min. When subjected to both *stimuli*, M07e cells were incubated with MBCD, washed and treated with SCF. Cell suspensions were washed with PBS, then [^3^H]-cholesterol content was estimated by liquid scintillation counting. Results are expressed as means ± SD of four independent experiments, each performed in triplicate. **P<0.005, significantly different from control cells; ***P<0.0001, significantly different from control cells; ○○*P*<0.005, significantly different from the corresponding sample treated with SCF; ○○○*P*<0.0001, significantly different from the corresponding sample treated with SCF; §*P*<0.005, significantly different from the corresponding sample treated with MBCD.

In previous studies on cytokine-induced glucose transport stimulation performed in human leukemia M07e cells expressing mainly GLUT1 isoform [16], [25], we demonstrated that this acute effect occurs independently on the synthesis of new transporter molecules. Therefore, to investigate the influence of an altered bilayer cholesterol content on the GLUT1 activity, the effects of exposing cells to SCF and/or to cholesterol-depleting agent MBCD were compared. Cells treated with 10 mM MBCD for 20 min, washed and immediately tested for glucose uptake exhibited a high, significant rise in the glucose transport activity (Fig. 3A). No differences were observed when cells were subjected to the same treatment but assayed for glucose uptake after 40 min from the MBCD removal. This result allows to rule out, at least within 60 min, the involvement of a new cholesterol biosynthesis and/or the recruitment of free cholesterol from intracellular esters.

The rise observed in MBCD-treated cells was as high as that obtained in SCF-treated cells, and the combined treatment of M07e cells with both MBCD and SCF caused a further, significant increment in this uptake, showing an additive effect between the cytokine and the cholesterol-depleting agent. In addition, Fig. 3B shows the absence of SCF effect on the plasma membrane cholesterol content in the presence or absence of MBCD.

**Figure 4:**
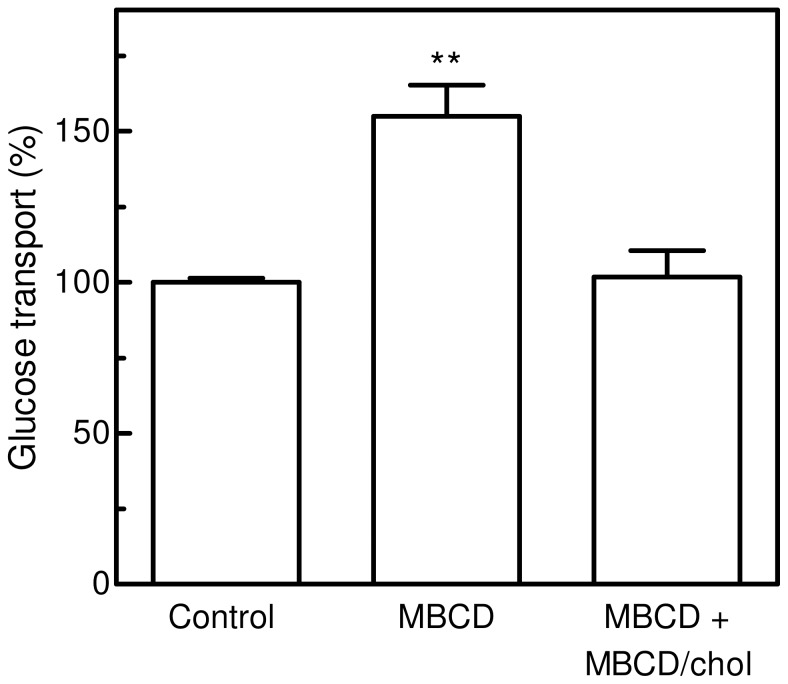
Effect of cholesterol replenishment on glucose transport in MBCD-treated M07e cells. IL-3-starved cells were incubated (or not) in PBS with 10 mM MBCD for 20 min, washed and re-suspended in PBS. A third sample of IL-3-starved cells was treated with MBCD for 20 min in PBS, washed and re-suspended in PBS, then added with MBCD/cholesterol mixture (25 mM cholesterol and 10% MBCD in PBS) for 40 min at 37°C, washed and re-suspended in PBS. Glucose uptake was assayed as described in the Materials and Methods section. Results are expressed as means ± SD of three independent experiments, each performed in triplicate. **P<0.005, significantly different from control cells.

To rule out a direct effect of MBCD on GLUT1 activity, we tried to replenish plasma membrane cholesterol content after the MBCD treatment (Fig. 4). The repletion procedure was accomplished by incubating cells in the presence of a MBCD/cholesterol mixture (25 mM cholesterol and 10% MBCD in PBS) for 40 min at 37°C. In fact, the high affinity of β-cyclodextrins for cholesterol can be used not only to remove it from biological membranes, but also to generate cholesterol inclusion complexes able to act as cholesterol donors [19]. The ratio between the amounts of cholesterol and cyclodextrin in the complex influences whether it will act as cholesterol acceptor or as cholesterol donor. Cholesterol replenishment did not affect the basal glucose transport activity of the cells (data not shown), while it abrogated the stimulatory effect observed in the presence of MBCD.

### Effect of Cholesterol Depletion on GLUT1 Distribution between Different Membrane Domains in M07e Cells

**Figure 5:**
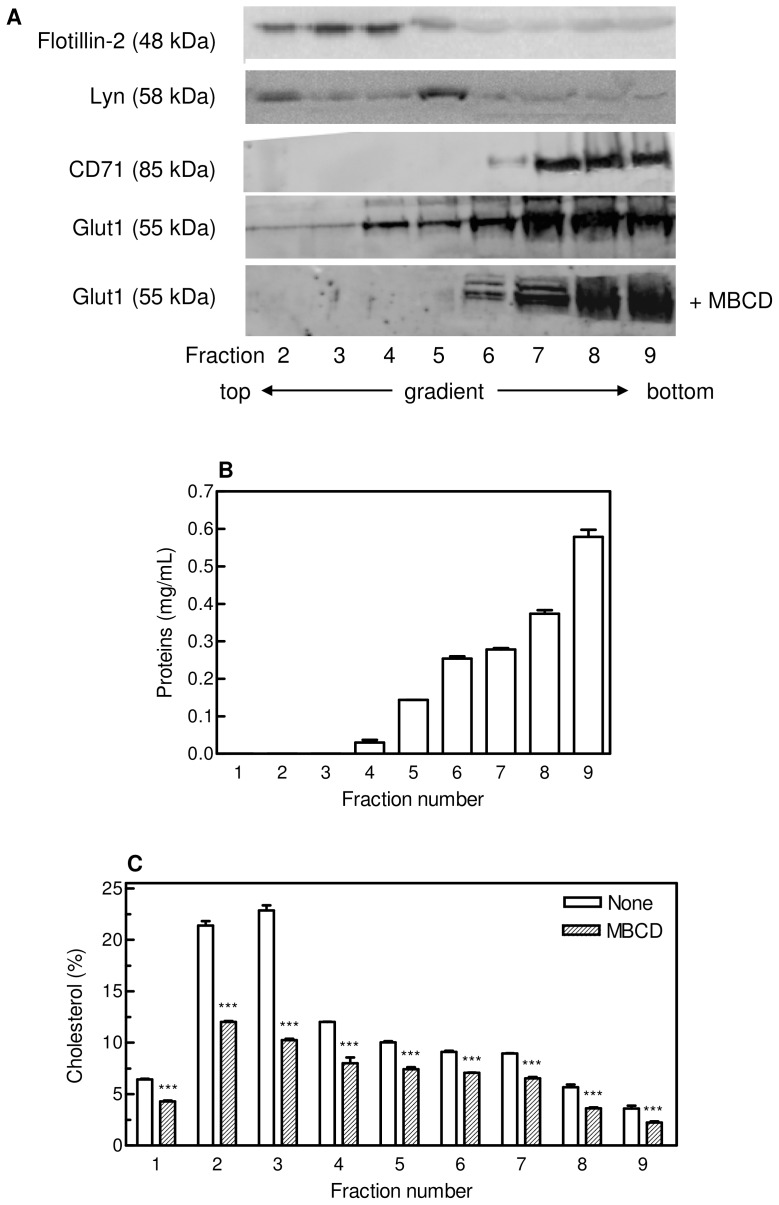
GLUT1 and cholesterol distribution along sucrose-gradient fractionation of M07e cells treated or not with MBCD. (**A**) M07e cells treated (or not) with 10 mM MBCD for 20 min were lysed with 1% Triton X-100 at 4°C and separated by sucrose density-gradient ultracentrifugation as described in the Materials and Methods section. Equal volume aliquots of each fraction were subjected to SDS-PAGE and Western blotting. Flotillin-2 and Lyn were used as markers for DRM fractions; CD71 for non-DRM fractions. (**B**) Typical profile of protein concentrations in gradient fractions after ultracentrifugation. Protein content was determined as described in the Materials and Methods section. (**C**) M07e cells were pre-incubated at 37°C for 16 hours with [^3^H]-cholesterol (0.1 µCi/mL) in cell culture medium. Cells exposed (or not) to 10 mM MBCD for 20 min were lysed with 1% Triton X-100 at 4°C and subjected to sucrose density-gradient ultracentrifugation as previously described. [^3^H]-cholesterol content of each fraction collected was quantified by liquid scintillation counting. Results are expressed as means ± SD of three independent experiments, each performed in triplicate. ***P<0.001, significantly different from untreated cells.

The presence of caveolae/lipid rafts in unstimulated M07e cells was recently reported [26], thus we isolated these domains by flotation on sucrose density gradient, in order to test whether changes in the lipid environment surrounding GLUT1 are associated with its distribution between different microdomains of the plasma membrane. M07e cells were lysed and separated by sucrose density-gradient (5–40%) centrifugation as described in the Material and Methods section. Nine fractions were collected, and aliquots were analyzed by SDS-PAGE followed by Western Blotting. The proteins flotillin-2 (48 kDa) and Lyn (58 kDa) are known to be associated with lipid rafts/caveolae in a variety of cells and so they were used as markers of DRM fractions [13]; transferrin receptor (CD71, 85 kDa), an integral membrane protein, was considered a marker for non-raft membrane fractions. As it can be seen in Fig. 5A, DRMs from untreated cells were localized in the low-density region of the gradient (fractions 2–5), between approximately the 10% and 25% sucrose layers. GLUT1 protein is distributed along the gradient, being more abundant in the high-density regions (fractions 6–9), but showing also a co-localization with flotillin-2 and Lyn in fractions 2–5. When cells were treated with an amount of MBCD able to rise the glucose uptake to about 60%, the distribution profile of GLUT1 along the gradient was changed, and the glucose transporter resulted totally confined to the high-density region on the gradient. This GLUT1 shift could be involved in the observed glucose transport stimulation obtained upon MBCD treatment. Fig. 5B represents the protein content of the different gradient fractions and shows that the bulk of M07e protein was found in the high-density region at the bottom of the sucrose gradient. Moreover, since it has been shown that MBCD is capable of removing cholesterol from both raft and non raft fractions [27], we investigated the effect of MBCD treatment on cholesterol distribution in sucrose gradient fractionation in our experimental conditions. M07e cells were labelled with [^3^H]-cholesterol as described in the Material and Methods section. Cells were then exposed (or not) to 10 mM MBCD for 20 min, lysed with Triton X-100 at 4°C and subjected to sucrose gradient centrifugation. As reported in Fig. 5C, fractions 2–5, where DRMs are localized, exhibited the highest tritiated cholesterol content. Moreover, the cholesterol distribution profile of the samples treated with MBCD shows that, in our experimental conditions, fractions 2 and 3 exhibited an higher cholesterol depletion, evidencing a more efficient cholesterol removal from DRMs compared to the other fractions.

### Effect of MBCD on GLUT1 Translocation from Intracellular Vesicles to Plasma Membrane

**Figure 6:**
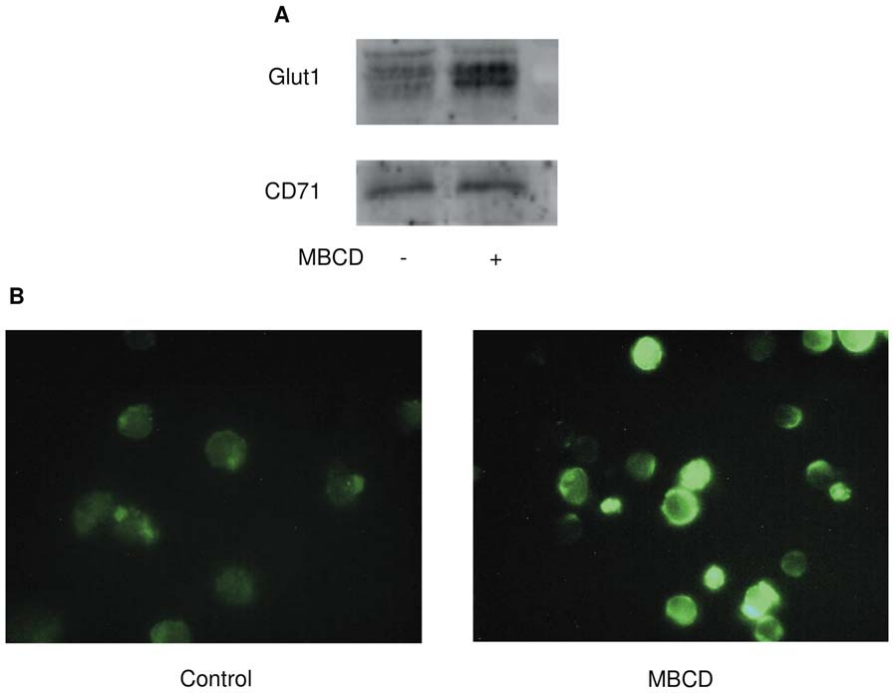
Enrichment of plasma membrane GLUT1 content in MBCD-treated M07e cells. (**A**) M07e cells were incubated (or not) in PBS at 37°C with 10 mM MBCD for 20 min. To isolate plasma membranes, cells were treated with NHS-LC-biotin; the mixtures were then added with streptavidin-agarose beads and the samples subjected to SDS-PAGE and immunoblotting with anti-GLUT1 as described in the Materials and Methods section. CD71, a plasma membrane protein marker, was used as a control. (**B**) M07e cells incubated (or not) in PBS at 37°C with 10 mM MBCD for 20 min, were fixed in 3% (w/v) paraformaldheyde for 15 min. Cells were then immunolabelled with anti-GLUT1 (N-20) antibody (raised against an extracellular domain of GLUT1), treated with fluorescent FITC-conjugated secondary antibody and visualized using immunofluorescence microscopy.

To better understand the effect of MBCD on the relative GLUT1 content in plasma membranes, we used a mild cell surface biotinylation to separate cell membrane fraction from cytosolic fraction. As reported in Fig. 6A, immunoblotting shows a significant increase of the amount of GLUT1 protein in plasma membranes following the treatment of M07e cells with 10 mM MBCD for 20 min. These data have been confirmed by immunofluorescence microscopy (Fig. 6B). Examination of cells labelled with anti-GLUT1 antibodies revealed that incubation with 10 mM MBCD for 20 min greatly enhanced the staining for the transporter at the cell surface. These results confirm that, in M07e cells, activation of glucose transport by MBCD could involve a GLUT1 translocation from intracellular pools.

### Effect of Phloretin or Nystatin on GLUT1 Activity

**Figure 7:**
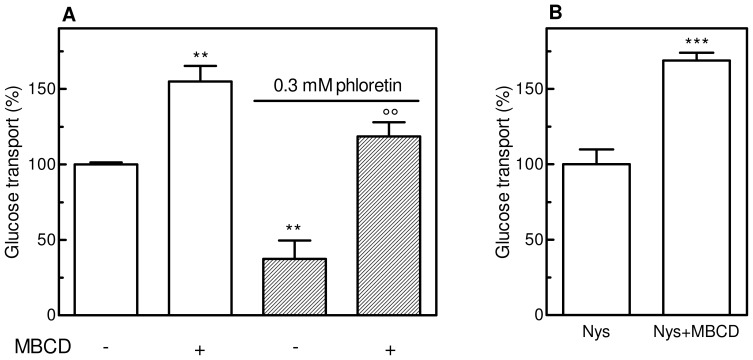
Effect of phloretin and nystatin on glucose transport in M07e cells with or without MBCD. (**A**) IL-3-starved cells were incubated (or not) in PBS at 37°C with 10 mM MBCD for 20 min, washed and re-suspended in PBS prior to the measurement of glucose transport as described in the Materials and Methods section (empty bars). A second batch of IL-3-starved cells was added with 0.3 mM phloretin for 10 sec, washed and re-suspended in PBS. Cells were then incubated (or not) with 10 mM MBCD for 20 min at 37°C, washed and re-suspended in PBS prior to the measurement of glucose transport (striped bars). (**B**) IL-3-starved cells were incubated with 50 µg/mL nystatin (Nys) in cell culture medium for 3 h at 37°C, washed, re-suspended in PBS and treated or not with 10 mM MBCD for 20 min at 37°C, washed and re-suspended in PBS prior to the measurement of glucose transport as described in the Materials and Methods section. Results are expressed as means ± SD of three independent experiments, each performed in triplicate. **P<0.005, ***P<0.001, significantly different from control cells; ○○*P*<0.005, significantly different from the corresponding sample untreated with MBCD.

To better understand the mechanism of MBCD-induced glucose uptake enhancement, M07e cells were treated with phloretin, a specific inhibitor of glucose transporters that binds competitively to the exofacial glucose binding site [28]. M07e cells were exposed to the action of phloretin, washed, re-suspended in PBS, then added with DOG mixture. As shown in Fig. 7A, even though phloretin was washed out before glucose transport measurement, cells exhibited a remarkable decrease in glucose transport activity, suggesting that several phloretin molecules are still bound to the exofacial site of GLUT1. When cells subjected to the same treatment were incubated with 10 mM MBCD for 20 min prior to the glucose transport measurement, a significant, remarkable increase in glucose uptake was obtained. This result could be explained by the shift to the cell surface of new transporter molecules, coming from intracellular stores, thus having a free glucose binding site, since not affected by phloretin action.

To corroborate this observation, M07e cells were treated with 50 µg/mL nystatin (an endocytosis inhibitor) [21], in culture medium for 3 hours, washed, re-suspended in PBS, incubated or not with 10 mM MBCD for 20 min, washed and assayed for glucose transport activity. As shown in Fig. 7B, the pre-treatment of the cells with nystatin did not influence the increase in glucose transport rate induced by MBCD addition. However, MTT test performed in parallel revealed that nystatin treatment caused a significant decrease in the viability of M07e cells (data not shown).

### Effect of Tyrosine Kinase Inhibitors and Cholesterol Depletion on Glucose Uptake in M07e Cells

The additive effect of the combined SCF/MBCD treatment on glucose uptake shown in Fig. 3, suggests that the action of SCF and MBCD may proceed through two distinct mechanisms. Therefore, to identify some of the steps connecting SCF or MBCD *stimulus* to GLUT1 modulation and to highlight any potential differences, we tested the effect of two tyrosine kinase inhibitors, Imatinib mesylate, a c-kit inhibitor employed in the treatment of leukemia [29], and PP2, a potent inhibitor of the Src tyrosine kinase family [30], on glucose transport enhancement in M07e cells.

**Figure 8:**
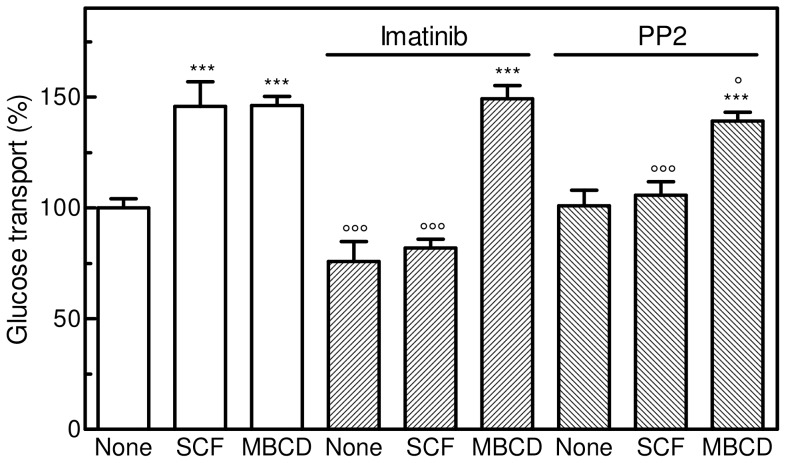
Effect of Imatinib or PP2 on glucose transport in SCF- or MBCD-treated M07e cells. IL-3-starved cells were incubated (or not) in PBS at 37°C with 5 ng/mL SCF for 15 min, then assayed for glucose transport activity as described in the Materials and Methods section. In the case of MBCD, cells were incubated in PBS at 37°C with 10 mM MBCD for 20 min, washed and re-suspended in PBS prior to the measurement of glucose transport. To test the effect of tyrosine kinase inhibitors, cells were pre-incubated with 10 µM Imatinib mesylate or 0.1 µM PP2 for 30 min at 37°C, washed and treated (or not) with SCF or MBCD as previously described and assayed for glucose transport activity. Results are expressed as means ± SD of three independent experiments, each performed in triplicate. ***P<0.0001, significantly different from the corresponding control cells; ○*P*<0.05, significantly different from the corresponding sample untreated with the inhibitor, ○○○*P*<0.0001, significantly different from the corresponding sample untreated with the inhibitor.

Fig. 8 shows that, in the presence of Imatinib mesylate, SCF-stimulated cells exhibited a significant reduction in glucose uptake activity, while in MBCD-treated cells this activity was unaffected. This result seems to indicate that the MBCD signaling pathway does not proceed through c-kit involvement. Moreover, glucose uptake in SCF-treated cells is significantly affected by the presence of PP2, while glucose transport enhancement of MBCD-treated cells is almost unchanged, suggesting that the MBCD signalling pathway does not proceed through Src kinase involvement.

### Phosphorylation Cascade Involved in the Modulation of Glucose Uptake Induced by SCF or MBCD

**Figure 9:**
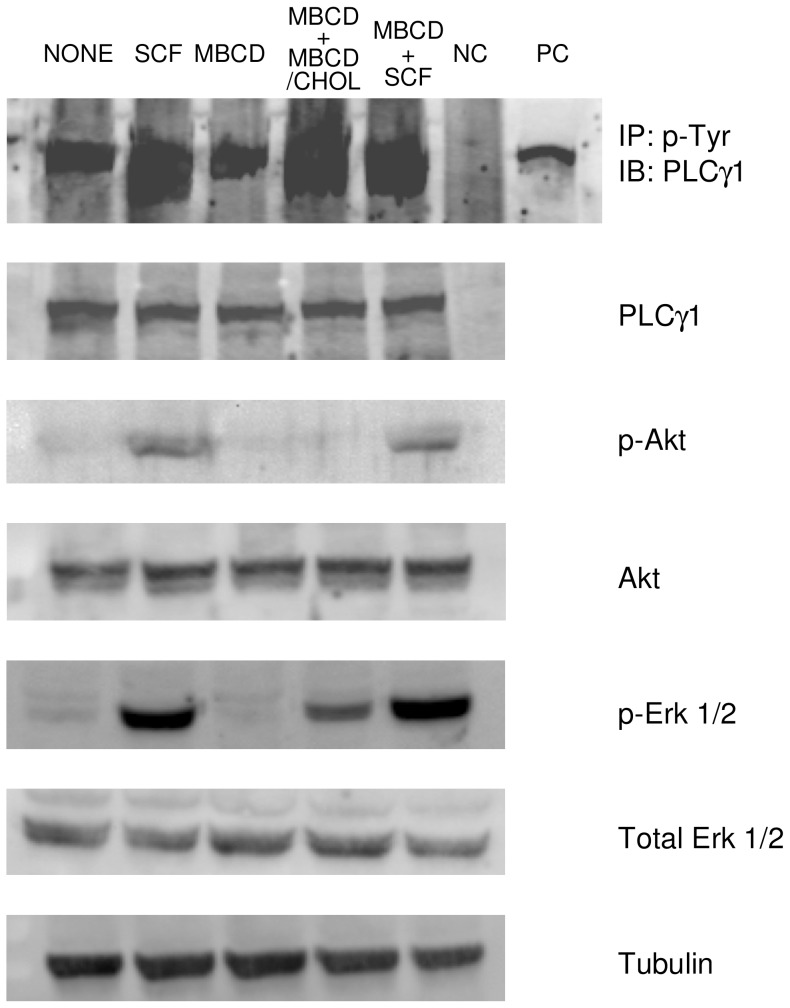
Effect of MBCD on Akt, PLCγ1 and ERK phosphorylation in M07e cells. IL-3-starved cells were incubated (or not) in PBS at 37°C with 10 mM MBCD for 20 min, or with 5 ng/mL SCF for 15 min. MBCD-treated cells were also added with MBCD/cholesterol mixture (25 mM cholesterol and 10% MBCD in PBS) for 40 min at 37°C, or 5 ng/mL SCF for 15 min. Cell lysates were electrophoresed and immunoblotted with the indicated antibodies as described in the Materials and Methods section. Tubulin detection was used as a control. NC: negative control; PC: positive control; IP: immunoprecipitation; IB: immunoblotting.

In a previous paper, the sequence of events leading to the glucose transport stimulation in response to SCF (and H_2_O_2_) was delineated [29]. The analysis of the effect of several kinase inhibitors suggested that the phosphorylation order downstream of c-kit activation can be as follows: Akt, PLCγ and Src. Since it has been reported that a direct link could exist between membrane cholesterol concentration and MAPK activation, we investigated also the presence of the phosphorylated forms of these enzymes in MBCD-treated M07e. To study the involvement of these kinases in the signaling pathway leading to the glucose transport enhancement induced by SCF or MBCD in M07e cells, we performed the Western blot analysis reported in Fig. 9. It can be seen that SCF-stimulated cells exhibited a remarkable increase of the phosphorylated form of Akt, PLCγ1 and ERK 1/2, while in MBCD-treated cells the level of these phosphorylated kinases was unchanged with respect to control cells. Cells pre-incubated in the presence of MBCD before the addition of SCF gave rise to the same effect observed in cells treated with SCF alone. Only in the case of ERK 1/2 cholesterol depletion seems to enhance the amount of activated form of these kinases induced by SCF. When M07e cells were subjected to MBCD treatment, then added with a MBCD/cholesterol mixture to replenish the depleted plasma membrane cholesterol, the phosphorylated forms of PLCγ1 and ERK 1/2 were also slightly increased (Fig. 9).

## Discussion

In this study we demonstrated that MBCD led to an increase in glucose uptake in M07e cells, mimicking the SCF effect. Both *stimuli* involved a subcellular redistribution of GLUT1, recruiting intracellular transporter molecules to the plasma membrane. We previously observed that SCF and H_2_O_2_ share the ability to promote GLUT1 translocation to the cell membrane through activation of the c-kit pathway [29]. On the contrary, data here reported show that cholesterol depletion is able to trigger GLUT1 translocation without the involvement of the c-kit signalling pathway and that the combined SCF/MBCD cell treatment on glucose uptake causes an additive effect. These observations suggest that the action of SCF and MBCD may proceed through two distinct mechanisms, the former following a signalling pathway, and the latter possibly being a nonsignaling, mechanical action. Data here reported suggest that MBCD exerts its action *via* a cholesterol-dependent mechanism that ultimately results in augmented GLUT1 translocation.

A relationship between plasma membrane cholesterol and glucose transporter GLUT1 has become apparent since a long time [11]. Studies on reconstituted human transporter demonstrated that small changes in bilayer cholesterol content result in drastic alterations in GLUT1 activity. These alterations appear to be primarily determined by bilayer composition rather than bilayer fluidity [11]. Changes in the cholesterol content of cell membrane might therefore be expected to affect the GLUT1 activity, as recently observed for GLUT4 isoform [9], [10].

Numerous studies have shown that cell exposure to β-cyclodextrins results in removal of cellular cholesterol; in particular, β-cyclodextrins have the highest affinity for inclusion of cholesterol and MBCD is the most efficient in extracting cholesterol from the membranes [18]. Best practice for the MBCD use includes the test of the degree of cholesterol depletion in one’s experimental conditions, because the efficiency of MBCD in extracting cholesterol may vary significantly depending on its concentration, duration of the exposure and the cell type. In our conditions, cell treatment with 10 mM MBCD for 20 min was able to remove about 60% cholesterol, establishing the desired mild lipid environment alteration associated with membrane integrity without affecting cell viability. Moreover, M07e cells were subjected to MBCD action in PBS buffer, where they can not obtain cholesterol from external sources (such as LDL present in FCS). Experiments reported in Fig. 3A show also that, in the chosen experimental conditions, cells have not enough time for a *de novo* cholesterol biosynthesis and, accordingly to the literature, they have a very low level of intracellular esters from which recruiting free cholesterol [31].

Several studies have shown that β-cyclodextrins are capable of removing cholesterol from both low and high density membrane fractions, suggesting that cholesterol is removed from both raft and non-raft fractions. Nevertheless, the efficiency of cholesterol removal may vary among different membrane domains [18]. It seems likely that the use of short time exposures or very low MBCD concentrations allows preferential depletion of cholesterol from lipid rafts [27]. Our results demonstrated that, although the cholesterol content of all membrane fractions was significantly reduced, in our experimental conditions MBCD was able to remove cholesterol more efficiently from DRMs.

Traditionally, lipid rafts have been isolated at 4°C using non ionic detergents and density-gradient ultracentrifugation. Here we used such a method to investigate whether the MBCD-induced re-distribution of the GLUT1 transporter between different membrane sub-domains might play a role in the modulation of its activity. The question as to whether rafts were a real physiological phenomenon or could be an artifact of the DRM preparation should be taken into account. In fact, it has been known that detergent solubilization is an artificial method which gives different results depending on the concentration and type of detergent, duration of extraction and temperature [15]. In line with these considerations, we used an alternative method to isolate raft-like membranes with a detergent-free medium containing a high concentration of sodium carbonate, obtaining similar results (data not shown).

Our results show that in sucrose gradient fractionation of lysed M07e cells, GLUT1 protein is more abundant in the high-density regions, while only a small portion is co-localized with lipid-raft marker proteins. In Clone 9 cells, Barnes et al. [13] observed that about the 36% of the total GLUT1 was located in the low-density region of the gradient, but it is conceivable that a variable portion of this transporter is associated with lipid-raft fraction depending on cell type. Following MBCD treatment, the glucose transporter resulted totally confined to the high-density region of the gradient, in agreement with data from Sakyo and Kitagawa, who reported that the insolubility of GLUT1 in Triton X-100 medium was reduced by cholesterol depletion [12].

It has been shown that in hemopoietic cells GLUT1 synthesis and glucose uptake are dependent on cytokine growth factors [3]. Previous reports from this laboratory have demonstrated that the hemopoietic cytokine SCF causes an acute stimulation of glucose transport in M07e cells upon 15 min treatment [25]. It is conceivable that, under these experimental conditions, a de novo synthesis of GLUT1 may be excluded, on the grounds that the time is not sufficient for a regulatory mechanism at transcriptional and/or translational level. Many mechanisms have been described for the enhancement in glucose transport rate occurring with a constant number of GLUT1 molecules: increased affinity of existing transporters in the plasma membrane [17]; translocation of GLUTs from intracellular pools to the plasma membrane, as shown for insulin-sensitive GLUT4 transporter [32], [33]; activation of GLUTs pre-existing in “masked” forms in the plasma membrane [34]. In insulin-responsive tissues, GLUT4 trafficking has been widely described, with the transporter cycling between the plasma membrane and one or more intracellular compartments, generally occurring through a PI3K-dependent pathway. Recently it has been reported the presence of a second, PI3K-independent, pathway proceeding through the involvement of lipid rafts. Biochemical and morphological techniques have revealed that these lipid domains contain several proteins involved in regulating GLUT4 translocation and glucose transport [32], [33]. Moreover, Liu and coworkers observed that exposure of 3T3-L1 preadipocytes to increasing concentrations of MBCD resulted in a dose-dependent stimulation of GLUT4 translocation [9].

Our data and results from other laboratories demonstrate that glucose transporters cell surface trafficking is not unique to GLUT4; it also occurs for GLUT1 in response to growth factors or oncogenic stimulation in noninsulin-responsive tissues [5], [6]. In M07e cells, the treatment with the cholesterol depleting agents stimulated glucose transport activity at the same extent obtained in SCF-treated cells and greatly enhanced the presence of GLUT1 at the cell surface, causing a transporter translocation from intracellular pools. Given the well known sensitivity of the glucose transporters to the lipid environment, we speculate that changes in plasma membrane lipid biochemistry can regulate GLUT1 recruitment to the plasma membrane, in a way similar to that observed with GLUT4. To corroborate our observations, we verified whether MBCD-induced GLUT1 activation could be prevented by restoring the basal state plasma membrane level of cholesterol. Cells incubated with MBCD/cholesterol inclusion complex prevented the glucose transport activation induced by MBCD, but did not alter its basal activity level. These findings demonstrate that the reduction of plasma membrane cholesterol content significantly influences GLUT1 activity. After MBCD treatment, GLUT1 content in plasma membrane could rearrange owing to an increase in exocytosis and/or a decrease in endocytic retrieval; experiments reported in Fig. 6 and 7 provide some evidences that the gain of GLUT1 in plasma membrane was due to an increase in exocytosis rather than a decrease in endocytic retrieval.

The additive effect of the combined MBCD/SCF treatment observed on glucose uptake of M07e cells suggests that the action of SCF and MBCD may proceed through distinct mechanisms. This hypothesis is supported also by the finding that SCF did not affect the plasma membrane cholesterol content in MBCD-treated cells.

We previously identified some of the steps connecting c-kit activation by SCF to GLUT1 modulation in M07e cells and demonstrated that Imatinib mesylate, a selective inhibitor of c-kit tyrosine kinase activity, and PP2, a potent inhibitor of Src family kinases, are able to block the glucose transport activation induced by SCF [29]. On the contrary, data here reported demonstrated that the glucose uptake of MBCD-treated cells was almost unaffected by the addition of Imatinib mesylate or PP2, indicating that MBCD signalling pathway does not proceed through c-kit involvement.

Since we have previously shown that the enhancement of the glucose transport activity observed in M07e cell line upon cytokine treatment is heavily dependent on the intracellular levels of ROS [25], we investigated whether MBCD treatment could induce ROS formation. As a probably consequence of MBCD disassembling action of lipid platforms, MBCD-treated cells exhibited a very low ROS production, allowing to exclude that MBCD-dependent glucose transport enhancement is related to an increase in ROS generation (data not shown).

Previous reports from this laboratory have demonstrated that in M07e cells the hemopoietic cytokine SCF causes an acute stimulation of glucose transport through a GLUT1 translocation from intracellular stores to plasma membranes and that this effect is mimicked by H_2_O_2_ [6]. Both *stimuli* are able to increase the phosphorylation of c-kit and this fact can explain why H_2_O_2_ mimics the SCF effect on glucose transport modulation [29]. In the same study we identified some of the steps connecting c-kit activation by SCF or H_2_O_2_ to GLUT1 modulation and demonstrated the involvement of the phosphorylated forms of Akt, PLCγ1, and Src, in this order.

Data here reported demonstrate that MBCD treatment fails to induce the phosphorylation of Akt and PLCγ1, indicating that in M07e cell line under the described experimental conditions, the c-kit-dependent SCF signaling pathway is not affected by the altered lipid microenvironment caused by MBCD addition. On the same cell line and under similar experimental conditions, Jahn and coworkers [26] observed similar results, but the use of higher MBCD concentrations resulted in a decrease of kit-dependent activation of Akt. The Authors suggest that c-kit localization in rafts is dynamic, depending on the ligand engagement and the duration time of its stimulation. Activation of c-kit tyrosine kinase is required for raft recruitment, but then raft recruitment is required for c-kit signalling. The mechanism for redistribution of the signalling molecules after c-kit activation is unknown, and it is suggested that could require the involvement of cytoskeleton. In our experimental system, it could be hypothesized that the exposure to 10 mM MBCD for 20 min is a condition that does not directly prevent c-kit activation and its downstream events, but it is sufficient to induce a GLUT1 translocation, which could be related to cytoskeleton alterations. In contrast to the cholesterol-dependent event being associated with a signal transduction mechanism per se, a non signalling basis may exist, therefore, it is possible that changes in raft properties may be coupled to actin cytoskeleton rearrangement. To this regard, a role for actin in insulin-stimulated GLUT4 translocation has been reported by several studies [9].

Cytokines are able to activate also ERK1/2, extracellular signal-regulated kinases involved in the regulation of mitosis and in postmitotic functions. Both Raf/MEK/ERK and PI3K/Akt/mTOR pathways are frequently activated in leukemia and other hematopoietic disorders caused by genetic mechanisms, playing an important role in the regulation of cell survival and proliferation [35].

Since it has been suggested that the MAPK pathway can be connected to the cholesterol level of caveolae [36], we investigated the presence of the phosphorylated forms of ERK enzymes in MBCD-treated M07e, showing that cholesterol depletion seems to enhance the amount of activated form of ERK induced by SCF. Our results are in agreement with those reported by Furuchi and Anderson [36], who observed that cholesterol depletion caused a marked increase in the amount of phospho-ERK in EGF-stimulated fibroblasts. They hypothesize that cholesterol depletion causes a disruption in the molecular organization of the MAP kinase signalling complex, which provokes a hyperactivation of the remaining caveolar ERK isoenzymes. They observed also that the hyper-responsive ERK in cholesterol-depleted caveolae is mitogenic, and this is in agreement with our data demonstrating that MBCD treatment slightly raises M07e proliferation (Fig. 2C).

The appearance of a small amount of the phosphorylated forms of PLCγ1 and ERK in M07e cells pre-incubated with MBCD and then treated with the mixture MBCD/cholesterol could be a consequence of perturbing plasma membrane cholesterol distribution, as observed by Casalou et al. about the activation of VEGF receptor 1 in acute leukemia cells [21]. In this paper a link between cholesterol and acute leukemias has been suggested, since cholesterol content in acute leukemia patient samples is higher than in healthy donor samples and correlates with disease aggressiveness. Other studies have reported that cholesterol uptake by leukemia cells promotes their survival and resistance to chemotherapy, leading to the use of statins (cholesterol-lowering agents) as therapeutic targets for subsets of acute leukemias, with reported clinical activity and efficacy [37]. Therefore, the findings reported in this study can concur to understand the role of cholesterol in cytokine-mediated signalling in acute leukemia cells, promoting the development of researches on focused therapeutic intervention based on the regulation of cholesterol metabolism and uptake.
